# Is There Any Overtrading in Stock Markets? The Moderating Role of Big Five Personality Traits and Gender in a Unilateral Trend Stock Market

**DOI:** 10.1371/journal.pone.0087111

**Published:** 2014-01-27

**Authors:** Jian Zhang, Haocheng Wang, Limin Wang, Shuyi Liu

**Affiliations:** 1 Department of business administration, Dongling School of Economics and Management, University of Science and Technology Beijing, Beijing, China; 2 Department of financial engineering, Dongling School of Economics and Management, University of Science and Technology Beijing, Beijing, China; Cinvestav-Merida, Mexico

## Abstract

Overtrading is a common anomaly among stock investors. This study examines the relationship between overtrading and investment returns and the impact of the Big Five traits and gender on overtrading in a unilateral trend stock market using a simulated stock investment system. The data were derived from a sample of undergraduates from six universities who performed in a simulated stock investment situation and had their personality traits measured by the Big Five Personality Questionnaire. The results indicate that: (1) Overtrading was significant in rising stock markets, but not significant in falling markets. (2) The degree of female investors who overtraded was significant in rising markets. (3) The degree of overtrading investors who were high in extroversion or agreeableness was significant in rising markets. The implications of these results for more effective investment strategies are discussed.

## Introduction

Overtrading is a common anomaly among stock investors, as observed in behavioral science research. As early as 1968, Jensen found that the returns of the most actively traded mutual funds were lower than the market rate of return [1]. Barber et al. [2] conducted empirical studies examining individual investor trading results in systematic and economically large losses using a complete trading history of all investors in Taiwan. Most researchers seem to support the existence of overtrading. However, other studies, such as that led by Hiemstra and Jones [3], determined a significantly positive relationship between trading volume and returns, which indicate that investors did not overtrade because the more they traded, the more they earned.

Do stock investors trade excessively? Does large trading volume reduce investment returns? Do market situations, personality and gender affect trading volume and thereby affect returns? Early studies were unable to reach an agreement on these questions. Insight into these issues would guide investors to adjust their investment behavior and increase their investment returns.

Studies investigating securities investment decisions are difficult to perform because an investment decision is similar to a ‘black-box’ in which the identity, asset scale, investing activity and return from a trade are sufficiently ambiguous that needed information is difficult to access. A number of researchers are able to obtain data from brokerage companies, but most of the information is dated. In the real market, the behavior of investors is affected by many factors, such as experience, knowledge and the market situation. These uncontrollable factors render research results unreliable. This article uses the simulated stock trading system developed by the University of Science and Technology Beijing to gain instant and accurate trading data. In the simulated system, the participants are allocated a certain amount of fictitious capital and stock that they can use to trade in a virtual network, thus affecting the price trend of the stocks. As the researchers can hide behind the network and act as a large institution to manipulate increases and decreases of stock prices, the real-world stock market can be simulated more closely. This type of simulated system is, by itself, an innovation in the study of experimental economics because it can accurately reflect the choices of participants when they make investment decisions. More importantly, the system allows variables to be manipulated experimentally and enables experimental conditions to be controlled, thereby ruling out unrelated factors and duplicate tests while simultaneously overcoming certain limitations of empirical studies.

### What is the Meaning of Overtrading?

In a conclusion that summarized the micro-level foundations of behavioral finance, DeBondt and Thaler [4] once noted that over-sized transactions in financial markets may be the most difficult to explain through traditional finance. By analyzing the transaction data of 66,465 investors in a brokerage company from 1991 to 1996, Barber and Odean [5] found that the annual return for those investors, whose transaction volume was comparatively large, was 11.4 percent, while the average market return was 17.9 percent. This result indicates that the phenomenon of overtrading exists and that a large number of transactions do not yield a higher return. Chen et al., [6] based on ten portfolios, found that stocks with the highest turnover of institutional ownership earn 8.9 percent lower subsequent one-year returns than stocks with the lowest turnover of institutional ownership. Tan & Wang [7] empirically researched the Chinese securities market and found that most of the trading decisions made by investors were wrong and that Chinese stock investors had the tendency to overtrade. After comparing the trading behavior of male and female investors, these authors found that male investors traded more excessively than female investors did. Barber and Odean [8] used account data for over 35,000 households from a large discount brokerage and analyzed the common stock investments of men and women from February 1991 through January 1997. These authors documented that men trade 45 percent more than women do. Trading reduced men’s net returns by 2.65 percent per year, as opposed to 1.72 percent per year for women. These authors also found that men trade more excessively than women do.

Drawing lessons from the above research, overtrading is defined in this paper by a negative relationship between trading volume and investment returns. When returns are significantly negatively influenced by trading volume, overtrading occurs; otherwise, overtrading is not present.

Is trading hazardous to one’s wealth? Our study addresses this issue by examining the relationship between trading volume and investment returns, as well as the influence of the investment situation, the Big Five personality dimensions and gender on overtrading. In this article, different investment situations were developed by manipulating stock prices, and the Big Five personality dimensions of participants were measured.

### Impact of Investment Situation on Trading Volume

In reality, the trading behavior of stock market investors is influenced by rising and falling markets. Shefrin and Statman [9] proposed a concept called the “disposition effect,” where investors tend to “sell winners too early and ride losers too long;” that is, investors are risk-averse when earning profits and are risk-embracing when suffering losses [10], therefore tending to sell stocks excessively in unilaterally rising markets and exhibiting reluctance to trade in unilaterally falling markets. Kahneman and Tversky [11] proposed a similar idea called the “prospect theory,” which stated that under uncertainty, people’s utility function was concave in the gains region and convex in the loss region, indicating that investors avoid risk in gains and embrace risk in losses.

These findings indicate that stock investors make different decisions when facing different investment situations, as measured by gains and losses. Specifically, when making decisions in possible gain situations, investors are risk-averse, selling large amounts of stocks when prices go up and becoming likely to trade excessively. In contrast, when making decisions in possible loss situations, investors tend to be risk-seeking and therefore do nothing when prices go down. The first hypothesis of this paper is the following:

### Hypothesis 1: Overtrading Exists in Situations with Unilaterally Rising Markets and Fades in Situations with Unilaterally Falling Markets

#### Impact of Big Five personality traits on trading volume

Previous studies explored the relationship between personality traits and investment behaviors using an experimental economics approach. Chinese scholars Peng & Wang [12] surveyed some stockholders in Shanghai concerning their investing behaviors and personality traits. They found that personality and temperament are important factors affecting investors’ behavior. Epstein & Garfield [13] were among the first researchers to divide investors into different personality types and explored the relationship between personality and investment returns. They noted that when a stock fits an investor’s personality, the investor could gain benefits. The Big Five personalities are considered to be present at the highest hierarchical level of individual behavior trait descriptions [14] and have considerable generalizability across languages and cultures [15]. A large amount of psychological research literature suggests that a Big Five personality dimension can predict leadership [16], job performance [17], [18], life outcomes/expectancy [19], academic achievement and years of education [20], and other social and economic results [21], [22]. This paper uses the Big Five personality dimensions to study the overtrading behavior of stock investors, which leads to a more reasonable forecast regarding the impact of personality on trading behavior.

#### Extroversion

Extroversion reflects the involvement of individuals in the outside world. Individuals with high extroversion scores are energetic and sociable, while individuals with low scores are independent and introverted [14], [23]. Applying these characteristics in the investment field, we suggest that investors with high extroversion scores are able to concentrate on reasonable investment decisions in spite of noise in the market. Furthermore, these individuals are likely to have good relationships with other people in the market; thus, they can access more information for investment decisions. Consequently, extroverted investors may be more rational and therefore less likely overtrade. Our first hypothesis related to the Big Five personality dimensions is the following:

### Hypothesis 2: Investors with High Extroversion Scores are Unlikely to Trade Excessively

#### Agreeableness

Agreeableness refers to individuals’ courteousness, trustworthiness, tolerance, compassion, generosity and cooperative demeanor [14], [15], [24]. Agreeableness measures an individual’s tendency toward cooperation, trust, tolerance, compassion, generosity and harmony. High-A individuals sometimes may be naive and can be misled by other people. Such individuals should improve their ability to question and analyze information, focus on details and develop persistence with regard to implementation. Excessive concerns and lack of independent thinking prevent high-A investors from making decisions decisively, resulting in herd behavior and high turnover in the market. Turnover in the Chinese stock market is sufficiently high that even in the recession year of 2002, the annual turnovers in the Shenzhen Exchange and the Shanghai Exchange were 198.9 percent and 214 percent, respectively [7]. Individuals with high-A scores would follow this behavior and trade excessively. Therefore, high-A investors tend to trade too much. Our second hypothesis related to the Big Five personality dimensions is the following:

### Hypothesis 3: Investors with High Agreeableness Scores are Likely to Trade Excessively

#### Conscientiousness

Conscientiousness reflects an individual’s tendency to act in an organized, effective, reliable and self-disciplined manner [14], [15], [24]. High-C individuals prefer to be realistic and leave work well arranged. Such individuals do not make decisions without adequate experience and information. As Behling [25] notes, conscientiousness is one of the best predictors of work performance. Furthermore, conscientiousness is also a powerful predictor of active, problem-focused response strategies [26], [27]. When making investing decisions, investors are bound to control and regulate their impulses and desires. High-C individuals are self-disciplined; thus, they control their desire to buy stocks with rising prices and sell stocks with falling prices. Therefore, high-C investors are reluctant to trade too much. The third hypothesis related to the Big Five personality dimensions is the following:

### Hypothesis 4: Investors with High Conscientiousness Scores will not Trade Excessively

#### Neuroticism

Neuroticism reflects an individual’s tendency to experience negative emotions; high-N individuals are apt to irrationally overreact to bad feelings and therefore experience psychological effects such as anxiety, pressure, hostility, tension, anger and depression [14], [23]. These individuals tend to be so sensitive and emotional that they overreact to ordinary situations and tiny frustrations. High-N investors focus on their feelings when making investment decisions; thus, once a stock price changes, they may experience extreme emotions, exhibit irrational behavior and trade too much. The fourth hypothesis related to the Big Five personality dimensions is the following:

### Hypothesis 5: Investors with High Neuroticism Scores are Likely to Trade Excessively

#### Openness

Openness is a personality dimension that represents the difference between conservative thought and open thought. It is the individual’s tendency to pursue novel, artistic, flexible and intellectual factors [14], [23]. High-O individuals are calm, independent and think critically in problem analysis. Such individuals are able to provide insight into the core of a problem. Wang et al. [28] from Peking University investigated 1,063 Chinese stockholders from seven cities in China and found that there were positive relationships between understanding an investment target, investment knowledge, independence, self-effectiveness and returns on investments. Investing in stock markets requires investors to collect information, make decisions accordingly and then to follow strategies. Investors with high openness scores have the advantage of access to information, and they are able to respond to changes in a volatile stock market. Because high-O investors are knowledgeable and intellectual [29], [30], they do not engage in overtrading. Our fifth hypothesis related to the Big Five personality dimensions is the following:

### Hypothesis 6: Investors with High Openness Scores are Reluctant to Trade Excessively Impact of gender on trading behavior

Previous research has noted that gender influences investors’ trading behavior; specifically, men behave over-confidently, while women do not [31], [32]. Lewellen, Lease & Schlarbaum [33] investigated the trading behavior of 972 investors and concluded that men have higher predicted stock prices and thus will trade more. Barber and Odean [8] also found that men trade more than women do by up to 45 percent. Meanwhile, Tan & Wang [7] investigated Chinese investors and documented that men have a higher tendency to trade excessively than do women. Therefore, the seventh hypothesis in this paper is the following:

### Hypothesis 7: Overtrading by Male Investors is more Significant than Overtrading by Female Investors

## Materials and Methods

### Experimental Platform

Experiments in this paper took place in the financial engineering laboratory of Dongling School of Economics and Management at the University of Science & Technology Beijing. This platform provided technical support for both hardware and software. The hardware included the servers and all the computers needed for the participants, while the software included the simulated stock trading system developed independently by the laboratory.

One server was used as the web interface server, and another server was used as the dealmaker server for the simulated stock trading system. Using the web interface server, participants in this experiment could determine their asset balance, make deals, delegate and revoke deals. The trading information then flowed into the dealmaker server, where buy and sell deals were matched according to the principle of price priority and time priority, and unmatched trading could be revoked by the participants.

### Trading System

In this research, the trading system used was a “T+0″(The settlement date of security transactions and denote that the settlement and delivery takes place immediately after the transaction occurs. To settle means to exchange the shares (stocks) for the cash and vice versa.) delivery and settlement system. Due to the experimental time limit and to set free the volatility of the stock prices and record the returns of investors with different risk attitudes, there were no tightening constraints on the price-changing margin, such as 5% or 10%. In this research, the limit of the price-rising margin was 500%, while the limit of the price-falling margin was 99.9%.

### Experimental Investment Situation

Unilateral movement is a common and simple trend in the stock market that indicates either a unilateral rising or a unilateral falling in stock prices. This research is based on this simple stock trend to analyze the impact of investment situations, Big Five personality dimensions and gender on overtrading behavior, and leaves the analysis of more complicated stock trends to further studies.

The experimental platform only allowed 160 subjects to be studied at one time, to ensure that data took on an apparent trend, the research was only designed with one stock. The number of shares issued, shareholders and the financial statements(Total Assets, Net Property, Cash Flow/Net Sales et al) information about the public company can be viewed in the simulation system. Participants can calculate the internal value(A stock’s internal value is the present value of its expected future cash flows to common shareholders, based on currently available information). Due to the experimental time limit and the main research is on the returns of stock trading, there are no dividends. Each of the participants in each of the experimental pre-openings was assigned ¥200,000 virtual cash and 10,000 shares of stock. To induce some variation across experiments, opening prices were different. After the opening, each test was traded freely at the same time by buyers and sellers; Each investment transaction time was 20 minutes, with a 5-minute break between each field being enacted by shutting down the system.

Before the experiment, all the participants were required to complete the Big Five personality assessment scale. Capital and stock were distributed evenly to each participant. The researcher then used a hidden account to manipulate the stock price and force the stock market to be unilaterally rising or falling, leaving the participant investors to make their own investing decisions and the computers to record their positions in trading volume and investment returns.

The basic principle for manipulation was to control the stock prices’ stability, or else the unilateral trend would be destroyed. Guided by this principle, some manipulations in these experiments took place in the third minute, while others took place in the fourth minute. Referring to the specific stock price trends, the manipulation in each experiment could form a rough trend because the stock price was also affected by the commissioned price paid by the participants, which was out of our control, as well as the stock trend determined by the equilibrium of price put forward by both buyers and sellers. Consequently, specific stock price trends could be manipulated by the researchers. Therefore, in this research, all stock price trends were treated equally, no matter the manner in which the trends were generated.

First, participants were provided with the following instructions: “These experiments will be conducted in a simulated stock investment situation, and the goal of the experiments is to study overtrading and the impact of personality type on the stock trading volume. You will be informed of the results at the end of the course. We designed one stock for trading in the simulation system. The number of shares issued, shareholders and information about the public company available in financial reporting statements can be viewed in the simulation system. Each student has been assigned ¥200,000 in virtual cash and 10,000 shares of stock. You can freely trade stocks after the beginning of the experiment. There are no dividends. The final trading returns will serve as the main reference for the grading of the investment course.” Then, participants conducted 6 experiments under non-identical conditions over the course of two weeks. The first three experiments were conducted in a unilaterally price-rising situation, while after two weeks, the following three experiments were conducted in a unilaterally price-falling situation.

To avoid the influence of the experimental market experience on the behavior of investors, the same participants conducted two further experiments with an inverted treatment order (falling prices first, then rising prices). Experimental platforms and parameters were the same as the previous experiment, with the only change occurring in the order of experimental situations. The first experiment was conducted in a unilaterally price-falling situation while the following was conducted in a unilaterally price-rising situation.

### Participants

The 115 participants in this experiment were students in the selected investment course at the University of Science and Technology Beijing; the students came from different specialized subjects from 16 universities. Among the participants, 43.5% were male and 56.5% were female. The students were permitted to communicate with one another and to trade online at the same time. Each experiment lasted 20 minutes, and there was a 5-minute break between each experiment. We employed standard psychological methods to study the relationship between personality and returns, which would not cause physical or psychological damage. We followed the guide of the Chinese Ethics Committee of Registering Clinical Trials and received the approval of Student Affairs, which was responsible for the safety of students and was a formal organization at the University of Science and Technology Beijing. Due to the number of students and because the students were online during the experiments, we only received the students’ oral consent; however, we also received the approval of the teacher in charge, and the students were informed that the purpose of this experiment was to study overtrading and the impact of personality and gender on trading volume before the experiment was begun, After the experiment, the results were shared with the students.

### Measures

#### Big Five personality

We used the five broad Big-Five personality domains (50 items) from International Personality Item Pool (IPIP) [34], [35], which uses 10 items to measure each of the five scales. Each item used a five-point scaled anchor, including strongly disagree, disagree, neutral, agree and strongly agree. IPIP scales are completely public domain; thus, no permission is required under any circumstances. The measure procedure also received the approval of Student Affairs at the University of Science & Technology Beijing. We used LISREL8.7 to verify the validity and deleted some items which did not satisfy some criteria(Factor loading>0.5), at last extroversion was measured using eight items (Cronbach’s α = 0.799), agreeableness was measured using seven items (Cronbach’s α = 0.780), conscientiousness was measured using eight items (Cronbach’s α = 0.796), neuroticism was measured using ten items (Cronbach’s α = 0.888) and openness was measured using seven items (Cronbach’s α = 0.798).

#### Trading variables

In this paper, trading volume was denoted as the number of stocks being traded by individual investors. The sum of the trading volume in the three experiments with a unilaterally rising stock price was treated as the trading volume in the investment situation with a unilateral increase in stock price. Similarly, the sum of the trading volume in the three situations with a unilaterally falling stock price was treated as the trading volume in the investment situation with a unilateral decrease in stock price. Meanwhile, the average return in each situation was treated as the return in the unilaterally price rising situation and the unilaterally price falling situation, respectively. The related formulas are:

TV in UPR = sum of TVs in three corresponding UPR experiments.

TV in UPF = sum of TV in three corresponding UPF experiments.

R in each experiment = (ending assets − beginning assets)/beginning assets.

R in UPR = average R in three corresponding UPR experiments.

R in DPI = average R in three corresponding UPF experiments.

TV stands for Trading Volume and R stands for Returns, while UPR stands for a Unilaterally Price Rising situation and UPF stands for a Unilaterally Price Falling situation.

### Statistical Analysis

The software used in the statistical analysis was SPSS19.0.

## Results

### The Price Processes and Timing of the Manipulations

**Figure 1:**
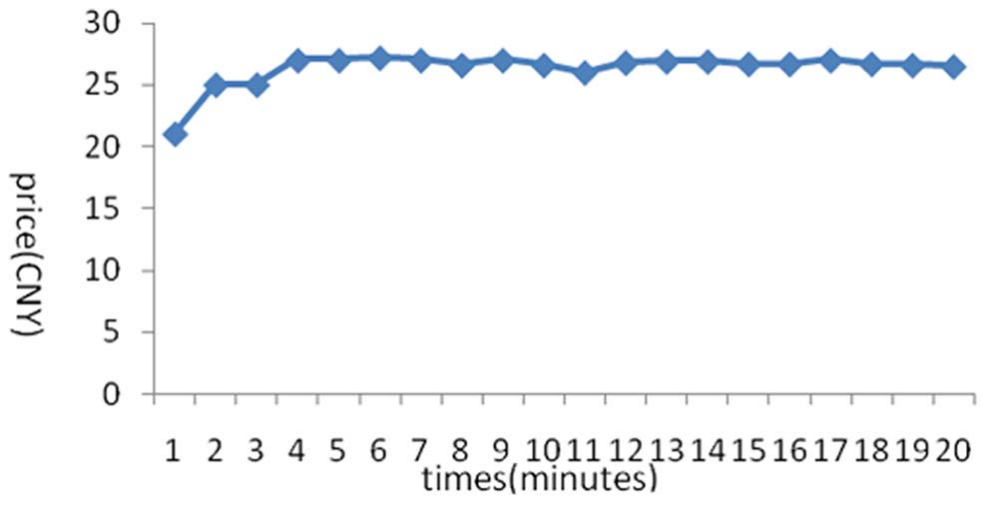
First Experiment of a Unilaterally Price-Rising Situation without Manipulation.

**Figure 2:**
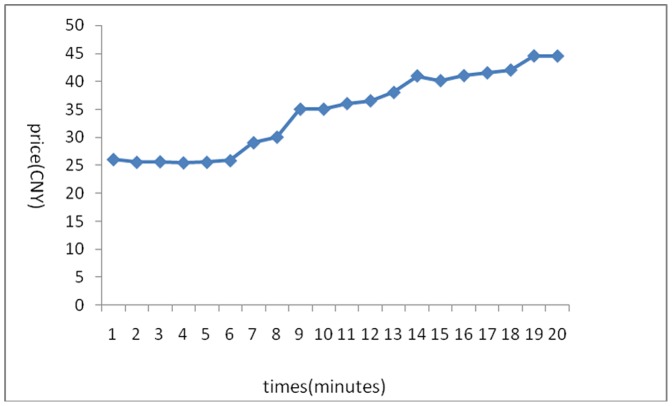
Second Experiment of a Unilaterally Price-Rising Situation with Manipulation.

**Figure 3:**
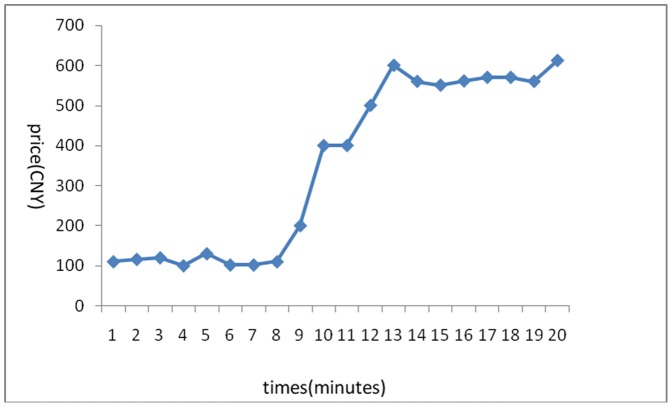
Third Experiment of a Unilaterally Price-Rising Situation with Manipulation.

**Figure 4:**
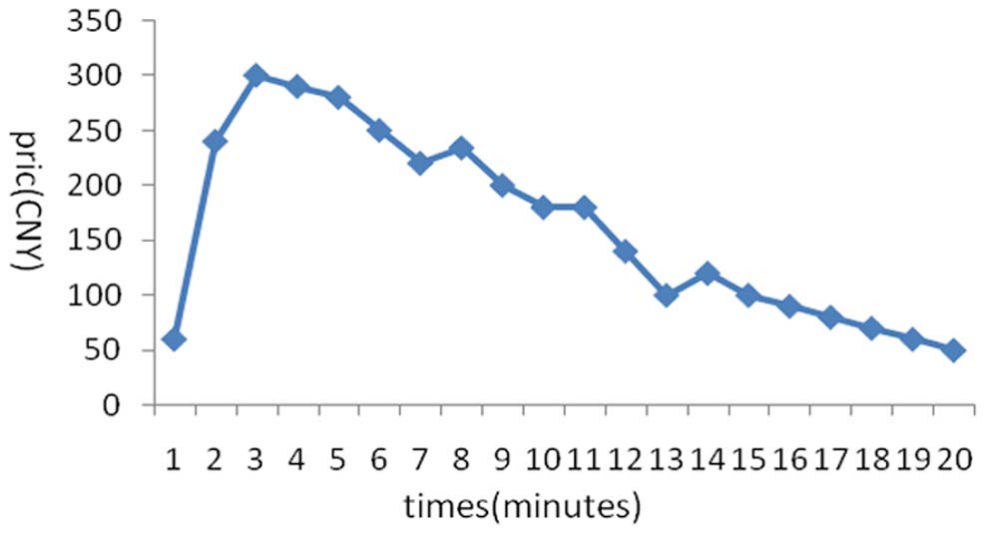
First Experiment of a Unilaterally Price-Falling Situation with Manipulation.

**Figure 5:**
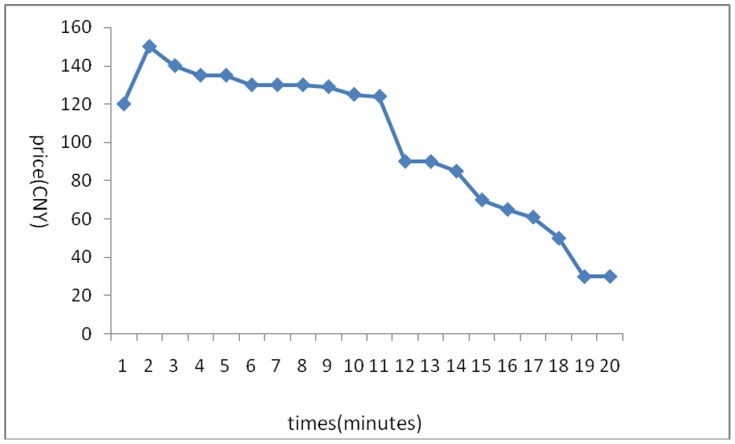
Second Experiment of a Unilaterally Price-Falling Situation with Manipulation.

**Figure 6:**
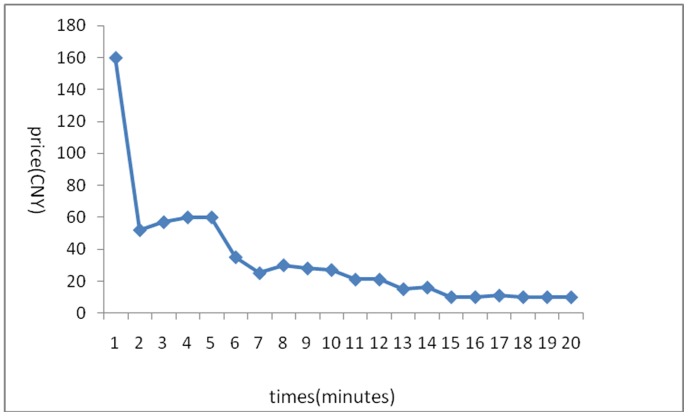
Third Experiment of a Unilaterally Price-Falling Situation with Manipulation.

The stock price trends generated in the 6 experiments are shown below:

Figure 1 reflects the stock price trends generated by the participants’ own behavior without any interference. It shows that in the early few minutes, the stock price rose because of a large amount of buy deals, but after it reached its peak, it fluctuated until the end of the experiments. Overall, the stock price trend was a steady rise without any manipulation. Figure 2 was generated by the setting that in the first three minutes, there was no manipulation, while afterward, the account held by the researchers began to buy stocks with higher and higher commissioned prices, leading to an intentional unilateral increase in stock price. Figure 3 shares a similar manipulation to that shown in Figure 2. The price trends generated in these two situations were similar, with a great increase in the stock price. An analysis of the data generated by these three experiments indicates that the stock returns were negatively related to trading volumes. Figure 4 reflects the situation of a falling price with manipulation where there was no interference in the first three minutes; the price kept rising naturally until it reached its peak, decreasing to a stable level in the fourth minute. At that moment, manipulation began by selling stocks at a low price, generating a unilaterally price-falling situation. The data analysis in this experiment beginning from the fourth minute shows that there was no significant relationship between stock returns and trading volume. To further justify this statement, we changed our manner of manipulation. Figure 5 was generated with a different manipulation setting where a soft manipulation started in the second minute and then a strong manipulation began in the tenth minute, leading to a unilateral decrease in price. Figure 6 shows a dramatic change from a high price to a low price, while in the last two minutes, the price had a rising trend, but it decreased again with another manipulation.

**Figure 7:**
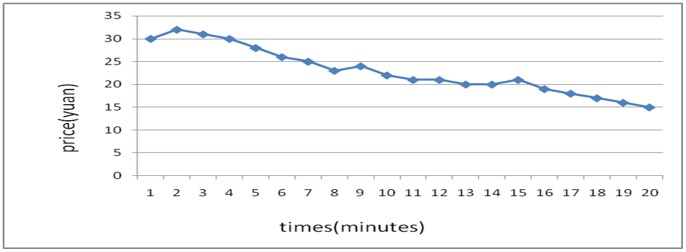
Experiment of a Unilaterally Price-Falling Situation with Manipulation.

**Figure 8:**
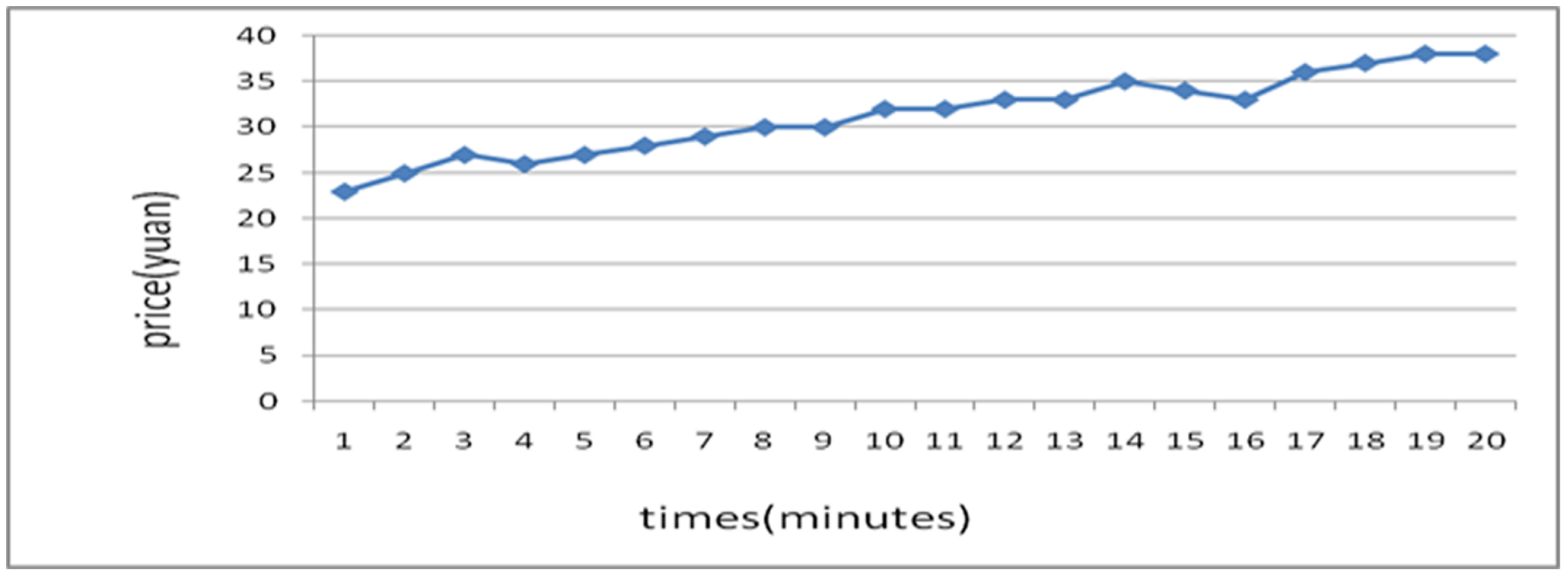
Experiment of a Unilaterally Price-Falling Situation with Manipulation.

The two experiments with an inverted treatment order (falling prices first, then rising prices) are shown below:

Figure 7 was generated with a strong manipulation in the beginning by selling stocks at a low price, leading to a unilateral decrease in price. Figure 8 was generated with a manipulation in the beginning by buying stocks with higher and higher commissioned prices, leading to an intentional unilateral increase in stock price. The result was that two experiments with an inverted treatment order (falling prices first, then rising prices) were taken on.

### The Relationship between Return and Trading Volume in Different Investment Situations

Descriptive statistics and the results of correlation analysis of returns and trading volume are presented in Table 1. The results indicate a negative relationship between returns and trading volume with a correlation coefficient of −0.303 (p<0.01) in unilaterally price-rising situations and an insignificant relationship between returns and trading volume with a correlation coefficient of 0.171 in unilaterally price-falling situations, supporting Hypothesis 1.

**Table 1:** Descriptive Statistics and Correlation Analysis of Returns and Trading Volume.

Variables	M	SD	1	2	3	4
TV in UPR	12360.81	15994.40	1			
TV in UPF	9900.00	7820.00	.286*	1		
R in UPR	.36	.35	−.303**	−.204	1	
R in UPF	−.29	.20	−.196	.171	.192	1

NOTE: TV stands for Trading Volume, R stands for Returns, UPR stands for Unilaterally Price Rising situation and UPF stands for Unilaterally Price Falling situation.

* *p*<0.05,

** *p*<0.01.

Descriptive statistics and the results of correlation analysis of returns and trading volume with respect to the inverted order treatment are presented in Table 2, which corresponds to the further experiments to balance the order effect with an inverted treatment order (falling prices first, then rising prices). The results indicate a negative relationship between returns and trading volume with a correlation coefficient of −0.212 (p<0.01) in unilaterally price-rising situations and an insignificant relationship between returns and trading volume with a correlation coefficient of −0.017 in unilaterally price-falling situations, also supporting Hypothesis 1. The results show that overtrading occurred in unilaterally price-rising situations, while in unilaterally price-falling situations, no overtrading occurred.

**Table 2:** Descriptive Statistics and Correlation Analysis of Returns and Trading Volume in an inverted treatment order experiment.

Variables	M	SD	1	2	3	4
TV in UPR	5297.87	13956.52	1			
TV in UPF	3944.68	5485.61	.346*	1		
R in UPR	.96	1.35	−.212*	−.063	1	
R in UPF	−.41	.23	−.045	−.017	−.564**	1

NOTE: TV stands for Trading Volume, R stands for Returns, UPR stands for Unilaterally Price Rising situation and UPF stands for Unilaterally Price Falling situation in an inverted treatment order experiment.

* *p*<0.05,

** *p*<0.01.

### The Impacts of Personality and Gender on Trading Volume

Descriptive statistics and correlation analysis of personality and gender on trading volume are shown in Table 3. These rates indicate an insignificant relationship between trading volume and the Big Five personality dimensions in both the unilaterally rising and the unilaterally falling scenarios, while trading volume is significantly influenced by gender. The results of the T-tests used to analyze the relationship between gender and trading volume are shown in Table 4. Table 4 shows that the trading volumes of male investors are higher than those of female investors in both unilaterally price-rising and unilaterally price-falling situations.

**Table 3:** Descriptive Statistics and Correlation Analysis.

Variables	M	SD	1	2	3	4	5	6	7	8
Extroversion	26.79	5.35	1							
Agreeableness	26.85	4.37	.355**							
Conscientiousness	27.32	6.01	.019	.286**						
Neuroticism	32.34	9.09	−.008	−.290**	.658**					
Openness	24.66	4.74	.644**	.285**	−.030	−.018				
Gender	.59	.50	.224*	.169	.003	.051	−.095			
TV in UPR	9900.00	7820.00	.000	.056	−.086	−.042	.138	−.401**		
TV in UPF	12360.81	15994.40	−.114	.088	.136	−.122	.107	−.370**	.286**	1

NOTE: The code of gender (1 = women, 0 = men). TV stands for Trading Volume, R stands for Returns, UPR stands for Unilaterally Price Rising situation and UPF stands for Unilaterally Price Falling situation.

* *p*<0.05,

** *p*<0.01.

**Table 4:** T-test of the effect of gender on trading volume.

Market situations	Gender	M	T	Sig
UPR	Man	12933.33	3.658	.000
	Female	6691.23		
UPF	Man	14807.40	2.392	.019
	Female	6907.97		

NOTE: UPR stands for Unilaterally Price Rising situation and UPF stands for Unilaterally Price Falling situation.

Table 3 shows that the Big Five personality dimensions do not affect individual trading volumes. However, because Table 1 showed a significantly negative relationship between returns and trading volume in unilaterally price-rising situations, further analysis of the impact of personality and gender on trading volume should be undertaken. The regressions were conducted for two subgroups composed of members with either high (1 SD above the mean) or low (1 SD below the mean) [36] personality scores in unilaterally price-rising situations, which are shown in Table 5. The table shows a significantly negative relationship between returns and trading volume of female investors with a regression coefficient of −0.390 (p<0.05). No significant relationship was found for male investors, which is the opposite of the expected result indicated by Hypothesis 7. Regression coefficients for high-E investors and high-A investors were −0.69 (p<0.01) and −0.434 (p<0.05), respectively, which was significant enough to justify the relationship between returns and trading volume. However, other dimensions of personality do not show a significant relationship, indicating the opposite of the expected result indicated by Hypothesis 2, while simultaneously supporting Hypothesis 3 and not supporting Hypotheses 4 and 5.

**Table 5:** Regression of Returns on Trading Volume.

variable	Dependent variable(Returns in UPR)											
	Male	Female	High-E	Low-E	High-A	Low-A	High-C	Low-C	High-N	Low-N	High-O	Low-O
	N = 50	N = 65	N = 33	N = 34	N = 37	N = 35	N = 31	N = 34	N = 26	N = 29	N = 36	N = 37
Trading Volume	.059	−.390*	−.569**	−.046	−.434*	−.261	−.225	.077	−.207	−.304	−.304	−.166
*F*	.111	6.446*	6.833*	.023	5.877*	.658	.534	.066	.402	3.062	2.042	.226
*R^2^*	.003	.152	.324	.002	.255	.068	.051	.006	.043	.254	.254	.028

NOTE:UPR stands for Unilaterally Price Rising situation. High-E = Investors with high (1 SD above the mean) scores in extroversion; Low-E = Investors with low (1 SD below the mean) scores in extroversion; High-A = Investors with high (1 SD above the mean) scores in agreeableness; Low-A = Investors with low (1 SD below the mean) scores in agreeableness; High-C = Investors with high (1 SD above the mean) scores in conscientiousness; Low-C = Investors with low (1 SD below the mean) scores in conscientiousness; High-N = Investors with high (1 SD above the mean) scores in neuroticism; Low-N = Investors with low (1 SD below the mean) scores in neuroticism; High-O = Investors with high (1 SD above the mean) scores in openness; Low-O = Investors with low (1 SD below the mean) scores in openness.

* *p*<0.05,

** *p*<0.01.

## Discussion

The trading volumes of stock investors are always high, especially in the Chinese stock market. Because of these high trading volumes, many researchers assume that there is overtrading in the stock market. In this paper, we collected data from a simulated stock trading system and performed a statistical analysis on these data, finding that in unilaterally price-rising situations, there is a negative relationship between returns and trading volume. In other words, there was overtrading in the stock market. However, in unilaterally price-falling situations, there was no significant relationship between stock returns and trading volume, and overtrading did not occur. This result is consistent with Hypothesis 1, which stated that the investment situation would influence investing behavior, thus supporting the “disposition effect” and the “prospect theory” proposed by Shefrin and Statman [9] and Kahneman and Tversky [11], respectively. The disposition effect refers to investors who tend to sell a large amount of stock in unilaterally price-rising situations due to risk aversion, therefore trading too much, while in unilaterally price-falling situations, investors tend to hold stocks due to embracing risk, and do not trade as often.

The data analysis in this paper also indicates that investors’ trading volume is not influenced by the Big Five personality dimensions of investors. However, in unilaterally price-rising situations, the trading volume of an investor with high scores in extroversion and agreeableness could adversely predict stock returns. These results are consistent with Hypotheses 3, 4 and 6 but not Hypotheses 2 and 5. In Hypothesis 2, we hypothesized that extroverted investors may be more rational and therefore less prone to overtrading. However, the results show that investors with high extroversion scores trade excessively in unilaterally price-rising situations. Li and Liu [37] found that individuals with high extroversion scores show risk-seeking behaviors in different framework situations and that individuals with high extroversion scores may prefer to pursue risk and adventure. Thus, they could be predicted to tend to buy stocks at high prices and to trade excessively to release stock returns. However, in unilaterally price-falling situations, individuals with high extroversion scores may hold onto loses and not trade excessively. Similarly, in Hypothesis 5, we infer that investors with high neuroticism scores are likely to trade excessively. However, the results show that investors with high neuroticism scores do not trade excessively. Neuroticism reflects an individual’s tendency to experience negative emotions, and individuals with high neuroticism scores are apt to irrationally overreact to bad feelings. However, in the unilaterally price-rising situation, most individuals will gain returns and experience positive emotion. Thus, investors with high neuroticism scores could hold onto winning stocks and thus not trade excessively. In contrast, in the unilaterally price-falling situation, investors with high neuroticism scores could experience negative emotions encouraging them to sell losers early and therefore avoid incurring a large loss.

Regarding the analysis on gender’s impact on trading volume, we found that the trading volumes of male investors are higher than those of female investors in both unilaterally price-rising and price-falling situations. This result is consistent with previous research indicating that male investors are more confident than female investors and trade more than female investors do by up to 45% [31]–[33]. However, the results of our analysis show that in unilaterally price-rising situations, only the female investors’ trading volume affected the returns; that is, female investors are likely to trade too much, which is consistent with the findings of Shua et al [38] that female investors have a greater “disposition effect,” and they tend to sell more stocks than male investors do in unilaterally price-rising situations. Male investors are so confident that in unilaterally price-rising situations, they hold stocks that they believe will continue increasing. Therefore, in that case, a female investor would trade too much, while a male investor would not.

This paper focuses on the impact of the investment situation, personality and gender on overtrading. Due to the complexity of personality and the variety of factors affecting investment decisions in stocks, the conclusions from this research should be further investigated. There were some limitations experienced by researchers. First, the investment environment was in the simulated stock trading system. In the real world, there are so many types of stocks and investors that stock prices are also influenced by investment policy, company performance and the manipulation of institutions. Therefore, in a further study, more stocks should be included in the simulated system to reflect real-world stock markets more closely. Second, there were only 8 experiments that lasted 20 minutes with different manipulation methods, there are limitations to the external validity of the results. To confirm the relationships found in this paper, further research should include more experiments in different situations. Finally, the definition of overtrading in this paper involves the existence of a significantly negative relationship between returns and trading volume, ignoring the effect of market periods in the decision-making process of investors. Therefore, a time-series analysis should be utilized in further studies.

Despite these limitations, there are still some reasonable suggestions based on the conclusions in this paper. First, investors should hold appropriate stocks and gain returns in unilaterally price-rising situations because investors tend to sell winners too early and trade excessively in such situations. Second, female investors should decrease their trading volume and avoid early selling in unilaterally price-rising situations because they are likely to trade excessively. Finally, although personality as a whole does not affect trading volume, investors with high scores in extroversion and agreeableness tend to trade too much in unilaterally price-rising situations; thus, these investors should avoid pursuing risk and interference from other investors and decrease their trading volume at such times.

## Conclusions

Through analysis of the data collected in the simulated stock trading system, we observed that there is a significantly negative relationship between returns and trading volume in unilaterally price-rising situations, while there is no such relationship in unilaterally price-falling situations, which indicates overtrading in the former situation. The trading volume of male investors was significantly larger than that of female investors; however, males did not overtrade in unilaterally price-rising situations, while female investors did. Finally, there was no significant relationship between trading volume and the Big Five personality dimensions, but the returns of high-E investors and high-A investors were adversely affected by their trading volumes, which indicates overtrading by investors with high scores in extroversion and agreeableness.
